# Novel homozygous mutations in Pakistani families with Charcot–Marie–Tooth disease

**DOI:** 10.1186/s12920-021-01019-5

**Published:** 2021-06-30

**Authors:** Sumaira Kanwal, Yu JIn Choi, Si On Lim, Hee Ji Choi, Jin Hee Park, Rana Nuzhat, Aneela khan, Shazia Perveen, Byung-Ok Choi, Ki Wha Chung

**Affiliations:** 1 Department of Biosciences, COMSATS University Islamabad, Sahiwal, Pakistan; 2 Department of Biological Sciences, Kongju National University, 56 Gongjudaehakro, Gongju, 32588 Korea; 3 Department of Pediatric Neurology, The Children Hospital and Institute of Child Health, Multan, Pakistan; 4 Department of Zoology, The Women University, Multan, Pakistan; 5 Department of Neurology, Samsung Medical Center, Sungkyunkwan University School of Medicine, 81 Irwon-ro, Gangnam-gu, Seoul, 06351 Korea

**Keywords:** Charcot–Marie–Tooth disease (CMT), Consanguinity, Homozygosity, Pakistan, Whole exome sequencing

## Abstract

**Background:**

Charcot–Marie–Tooth disease (CMT) is a group of genetically and clinically heterogeneous peripheral nervous system disorders. Few studies have identified genetic causes of CMT in the Pakistani patients.

**Methods:**

This study was performed to identify pathogenic mutations in five consanguineous Pakistani CMT families negative for *PMP22* duplication. Genomic screening was performed by application of whole exome sequencing.

**Results:**

We identified five pathogenic or likely pathogenic homozygous mutations in four genes: c.2599C > T (p.Gln867*) and c.3650G > A (p.Gly1217Asp) in *SH3TC2*, c.19C > T (p.Arg7*) in *HK1*, c.247delG (p.Gly83Alafs*44) in *REEP1*, and c.334G > A (p.Val112Met) in *MFN2*. These mutations have not been reported in CMT patients. Mutations in *SH3TC2*, *HK1*, *REEP1*, and *MFN2* have been reported to be associated with CMT4C, CMT4G, dHMN5B (DSMA5B), and CMT2A, respectively. The genotype–phenotype correlations were confirmed in all the examined families. We also confirmed that both alleles from the homozygous variants originated from a single ancestor using homozygosity mapping.

**Conclusions:**

This study found five novel mutations as the underlying causes of CMT. Pathogenic mutations in *SH3TC2, HK1*, and *REEP1* have been reported rarely in other populations, suggesting ethnic-specific distribution. This study would be useful for the exact molecular diagnosis and treatment of CMT in Pakistani patients.

## Background

Charcot–Marie–Tooth disease (CMT) and related neuropathies are a group of genetically and clinically heterogeneous peripheral neuropathies with a prevalence of approximately 1 in 2500 people [1]. CMT, also called hereditary motor and sensory neuropathy (HMSN), involves the impairment of both sensory and motor nerves, whereas distal hereditary motor neuropathy (dHMN) and hereditary sensory and autonomic neuropathy (HSAN) affect only motor and sensory nerves. Their common clinical phenotypes include progressive distal muscle weakness and atrophy, loss of sensation, and hyporeflexia of the upper and lower limbs [2]. CMT is commonly divided into demyelinating type (CMT1) with a reduced motor nerve conduction velocity (NCV) of less than 38 m/s, axonal type (CMT2) with normal or slightly reduced NCV of 38 m/s or more, and intermediate CMT type (Int-CMT) with NCV values spanning both CMT1 and CMT2 in a single family [3, 4]. CMT is often viewed as a monogenic Mendelian disease; however, mutations in more than 130 genes are associated with the development of peripheral neuropathies in an autosomal or X-linked dominant or recessive manner.

Several studies have attempted to determine the underlying causes of CMT [5]. In particular, the use of next generation sequencing (such as whole exome or targeted sequencing) has enhanced the identification of genetic pathogenicities. However, only a few studies have been performed to determine the genetic causes of CMT and related peripheral neuropathies in Pakistan [6–9]. Pakistani patients for whom genetic causes were identified exhibited unusually high frequencies of recessive homozygous mutations. Pedurupillay et al. reported three patients with CMT2S or spinal muscular atrophy with respiratory distress type 1 (SMARD1) with *IGHMBP2* mutations [7]. Two of them presented homozygous mutations. Wright et al. reported a homozygous *FIG4* variant in four independent patients manifesting combined phenotypes of CMT4J and Yunis‐Varón syndrome [9]. Houlden et al. reported several patients with *HSPB1* mutations which contained a homozygous mutation in addition to heterozygous mutations [6]. Zambon et al. reported a patient with CMT4B1 with homozygous *MTMR2* mutation [8]. The high rates of homozygous mutations in Pakistani patients can be attributed to the relatively frequent occurrence of consanguineous marriages.

This study aimed to determine the genetic causes of CMT or related neuropathies using in Pakistani patients using whole exome sequencing (WES) and subsequent filtering of called variants. We identified five pathogenic or likely pathogenic homozygous mutations in CMT-related genes. Additionally, we determined that all the observed homozygous mutations originated from a single founder through homozygosity mapping.

## Methods

### Subjects

**Fig. 1:**
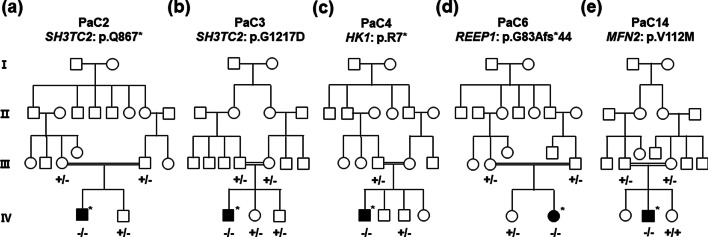
Five Pakistani consanguineous pedigrees with autosomal recessive CMT. Genotypes of pathogenic or likely pathogenic mutations are indicated at the bottom of all the examined family members. Black and white symbols represent affected and unaffected individuals, respectively. The affected individuals subjected to whole exome sequencing are indicated by an asterisk (□: male, and ○: female). **a** PaC2 family with c.2599C > T (p.Gln867*) in *SH3TC2*, **b** PaC3 family with c.3650G > A (p.Gly1217Asp) mutation in *SH3TC2*, **c** PaC4 family with c.19C > T (p.Arg7*) mutation in *HK1*, **d** PaC6 family with c.247delG (p.Gly83Alafs*44) mutation in *REEP1*, and **e** PaC14 family with c.334G > A (p.Val112Met) mutation in *MFN2*

This study examined five CMT patients and 15 of their unaffected familial members originating from consanguineous Pakistani pedigrees who were negative for the duplication or deletion of the 17p12 chromosomal region harboring the *PMP2*2 gene (Fig. 1). All participants were recruited from The Children Hospital and Institute of Child Health, Multan, Pakistan, and provided written informed consent. For the minors involved in the study, written consent was provided by their parents. This study was approved by the Institutional Review Boards for Kongju National University (KNU_IRB_2018-06), Sungkyunkwan University, Samsung Medical Center (2014-08-057-002), COMSATS University Islamabad, Sahiwal (CUI-SWL-09-062-2018), and The Children Hospital and Institute of Child Health, Multan (CHMP-2018-023-0014).

### Clinical and electrophysiological assessments

Motor and sensory impairments, deep tendon reflexes, and muscle atrophy were measured as the clinical information. Onset age was determined by interviewing the patients about when the symptoms, such as distal muscle weakness, foot deformity, or sensory change first appeared. Disease severity was determined using the functional disability scale (FDS) [10]. Motor and sensory conduction velocities of the median, ulnar, peroneal, tibial, and sural nerves were determined by surface stimulation followed by recording using electrodes. Motor nerve conduction velocities (MNCVs) of the median and ulnar nerves were determined by stimulating at the elbow and wrist while recording compound muscle action potentials (CMAPs) over the abductor pollicis brevis and adductor digiti quinti, respectively. In the same way, the NCVs of peroneal and tibial nerves were determined by stimulation at the knee and ankle while recording CMAPs over the extensor digitorum brevis and adductor hallucis, respectively. Sensory nerve conduction velocities (SNCVs) and sensory nerve action potential (SNAP) amplitudes were obtained over a finger-wrist segment from the median and ulnar nerves by the orthodromic method and were also recorded for sural nerves. Electromyography was carried out with a concentric needle electrode. Magnetic resonance imaging (MRI) of the brain and spinal cord were carried out using a 1.5-T system (Siemens Healthineers, Erlangen, Germany).

### DNA purification and pedigree analysis

Genomic DNA was purified from whole blood using the HiGene Genomic DNA Prep Kit (Biofact, Daejeon, Korea). Paternity was confirmed for all the examined families by PCR amplification of STR markers using the PowerPlex Fusion System (Promega, Wisconsin-Madison, USA) followed by the resolution of the PCR products on a SeqStudio genetic analyzer (Life Technologies-Thermo Fisher Scientific, Foster City, CA, USA).

### Exome sequencing and filtering

WES was performed for patients in the five examined families. Exome was captured using the SureSelect Human All Exon 50 M kit (Agilent Technologies, Santa Clara, CA, USA), and sequencing was performed using the HiSeq 2000 Genome Analyzer (Illumina, San Diego, CA, USA). The UCSC assembly hg19 (GRCh37) was used as the reference sequence (http://genome.ucsc.edu). Small nucleotide variants (SNVs) were called using the GATK (https://software.broad institute.org/gatk/) and SAMtools (http://samtools.sourceforge.net/). Rare alleles with minor allele frequencies (MAFs) of < 0.01 were obtained from the 1000 Genomes Project (1000G, http://www.1000genomes.org/), and the Genome Aggregation Database (gnomAD. https://gnomad.broadinstitute.org/). Candidate variants for the genetic causes were also checked in the dbSNP (http://www.ncbi.nlm.nih.gov/snp) and ClinVar (https://www.ncbi.nlm.nih.gov/clinvar/). Pathogenicity of the variants was categorized into five grades (pathogenic, likely pathogenic, uncertain significance, likely benign, and benign) based on the guidelines of the American College of Medical Genetics and Genomics (ACMG) [11]. Pathogenic candidate variants were confirmed by Sanger sequencing using the SeqStudio genetic analyzers (Life Technologies-Thermo Fisher Scientific).

### In silico prediction and conservation analysis

In silico analyses to predict the mutation effect were performed using the programs of MUpro (http://www.ics.uci.edu/~baldig/mutation) [12], PolyPhen-2 (http://genetics.bwh.harvard.edu/pph2/) [13], PROVEAN (http://provean.jcvi.org/) [14], and Fahmm (http://fathmm.biocompute.org.uk/) [15]. Conservation analysis of the mutation sites was performed using the MEGA-X software, ver. 5.05 (http://www.megasoftware.net/). Genomic evolutionary rate profiling score (GERP) was determined using the GERP +  + program (http://mendel.stanford.edu/SidowLab/downloads/gerp/) [16].

### Homozygosity mapping

For the putative pathogenic homozygous variants, homozygosity mapping was performed to determine whether two alleles originated from a single founder. Homozygosity mapping was achieved through haplotyping of SNPs distributed around the corresponding mutations from the WES data of the affected persons using the method outlined by Park et al. [17].

## Results

### Clinical manifestations

In this study, we examined five consanguineous families with CMT. CMT types and clinical phenotypes are listed in Table 1.

**Table 1:** Clinical characterization of five Pakistani CMT patients

Item/patient (sex)	PaC2:IV-1 (male)	PaC3:IV-1 (male)	PaC4:IV-1 (male)	PaC6:IV-2 (female)	PaC14:IV-2 (male)
Gene: mutation	*SH3TC2*: p.Q867*	*SH3TC2*: p.G1217D	*HK1*: p.R7*	*REEP1*: p.G83Afs*44	*MFN2*: p.V112M
Type	CMT4C	CMT4C	CMT4G	dHMN5B/SMARD1	CMT2A2B
AOE/AOO (year)	6/3	10/3	11/1	2.5/ < 1	7/5
Muscle atrophy	Yes	Yes	Yes	Yes	Yes
FDS	3	3	3	3	3
Sensory loss	Yes	Yes	Yes	No	Yes
DTR, ankle	Absent	Absent	Absent	Decreased	Absent
Foot deformities	Yes	Yes	Yes	Yes	Yes
Brain/Spine MRI	ND	Normal brain	Normal spine	Normal brain	ND
Other symptoms	Scoliosis	Scoliosis, short stature	–	Mild respiratory distress	Vocal cord hoarseness
*Motor nerve conduction studies*					
Median CMAP (mV)	1.3	Absent	ND	Absent	ND
Median MNCV (m/s)	14.1	Absent	ND	Absent	ND
Ulnar CMAP (mV)	0.8	4.1	ND	Absent	ND
Ulnar MNCV (m/s)	12.8	25.0	ND	Absent	ND
Peroneal CMAP (mV)	0.8	Absent	ND	Absent	ND
Peroneal MNCV (m/s)	14.9	Absent	ND	Absent	ND
*Sensory nerve conduction studies*					
Median SNAP (μV)	Absent	10.6	ND	22.0	ND
Median SNCV (m/s)	Absent	26.0	ND	51.3	ND
Ulnar SNAP (μV)	Absent	ND	ND	22.2	ND
Ulnar SNCV (m/s)	Absent	ND	ND	39.5	ND
Sural SNAP (μV)	Absent	8.8	ND	16.3	ND
Sural SNCV (m/s)	Absent	32.0	ND	39.8	ND

AOE: age of examination, AOO: age of onset, CMAP: compound muscle action potential, DTR: deep tendon reflexes, FDS: functional disability scale, MNCV: motor nerve conduction velocity, ND: not done, SNAP: sensory nerve action potential, SNCV: sensory nerve conduction velocity

*indicates putative translational termination

In the PaC2 family, a 6-year-old boy was born with a full term pregnancy from healthy parents. He showed delayed development and frequent falls during walking since he was 3 years old. He did not complain of sensory symptoms; however, his vibration sensation was reduced. Deep tendon reflex at the knee was absent. Scoliosis and feet deformities were observed. No family history of such complaints was recorded. Motor nerve conduction studies revealed prolonged distal latencies and low distal CMAP amplitudes with no reproducible f-wave latencies and wave forms. He did not manifest SNAP responses in the upper and lower extremities. These findings were compatible with demyelinating CMT neuropathy.

In the PaC3 family, a 10-year-old boy born with full term from healthy normal parents had demyelinating CMT. At the age of 3, he was noticed to fall frequently and had difficulty standing from a sitting position. In addition to the CMT phenotype, he manifested scoliosis and short stature. Although he had difficulty in walking, he could walk unaided. Motor nerve conduction studies of median and peroneal nerves showed prolonged distal latencies and low distal CMAP amplitudes of median and peroneal nerves were absent, and those of ulnar and tibial nerves were decreased. Motor and sensory NCVs were decreased at the upper and lower extremities. Brain MRI showed no area of abnormal signal intensity.

In the PaC4 family, an 11-year-old boy born with full-term pregnancy from healthy parents. At the age of 15 months, he was unable to walk without support. His parents first noticed gait disturbance at the age of 2.5 years. No family history of such complaints was recorded. Neurological examination revealed decreased vibration and pain sensation, which was consistent with the results of a sensory nerve conduction study. Deep tendon reflex at the knee was absent, and feet deformities were observed. Lumbo-sacral spine MRI did not reveal any noticeable abnormal signals.

In the PaC6 family, a 2.5-year-old girl born full term from unaffected parents had congenital motor neuropathy. She exhibited delayed development. The affected girl manifested foot deformity and contractures of the distal phalanges before the age of 6 months, and her parents noticed neuromuscular defects before she was 1 year old. She had frequent falls during walking, and mild respiratory distress. Deep tendon reflex at the knee was decreased, and feet deformities were observed. No family history of such complaints was recorded. CMAPs of the median, ulnar, peroneal and tibial nerves were not evoked, but, normal SNAPs and SNCVs were observed in the sensory median, ulnar and sural nerves.

In the PaC14 family, a 7-year-old boy manifested axonal CMT with an onset of 5 years. He had vocal cord hoarseness as an additional symptom. He manifested delayed development. At the age of 5 years, he manifested gait disturbances and had frequent falls during walking. He did not complain of sensory symptom; however, his vibration and position sensation were reduced. Deep tendon reflex at the knee was absent, and feet deformities were observed. No family history of such complaints was recorded.

### Identification of novel homozygous pathogenic mutations

**Fig. 2:**
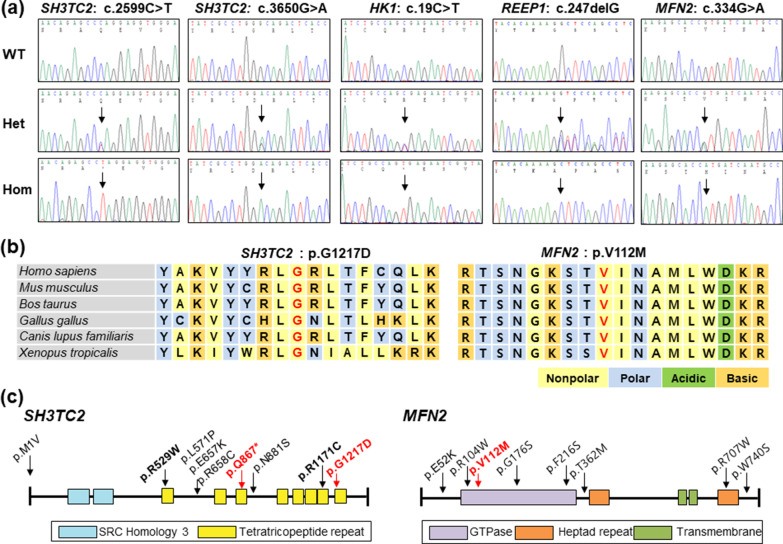
Identification of novel homozygous variants thought to be the underlying causes of CMT. **a** Sequencing chromatograms of c.2599C > T and c.3650G > A in *SH3TC2*, c.19C > T in *HK1*, c.247delG in *REEP1*, and c.334G > A in *MFN2*. **b** Conservation of two missense mutation sites. The amino acids at the mutation sites are highly conserved among vertebrate species. **c** Domain structure and location of the mutations of *SH3TC2,* and *MFN2*. The p.Gly1217Asp in *SH3TC2*, and the p.Val112Met in *MFN2* are located in the tetratricopeptide repeats (TPR) and GTPase domains, respectively

We identified five pathogenic or likely pathogenic homozygous mutations in the *SH3TC2, HK1, REEP1*, and *MFN2* genes in the examined families by sequencing analysis and rare allele  filtering processes (Table 2). All the candidate pathogenic mutations were confirmed by Sanger sequencing (Fig. 2a).

**Table 2:** Homozygous mutations and clinical phenotypes in five Pakistani CMT patients

Family ID	Gene	Mutation^a^	Type	Onset age (year)	Other symptom	Allele frequency		GERP	In silico prediction^b^				Note
						1000G	gnomAD		PP2	MU	PRO	Fath	
PaC2	*SH3TC2*	c.2599C > T;p.Q867*	CMT4C	3	Scoliosis	UR	UR	2.17	–	–	–	–	P
PaC3	*SH3TC2*	c.3650G > A;p.G1217D	CMT4C	3	Scoliosis, short stature	UR	1.6E−5	6.17	1.00*	0.10	− 5.79*	− 2.66*	P
PaC4	*HK1*	c.19C > T;p.R7*	CMT4G	1		UR	2.0E−5	1.13	–	–	–	–	P
PaC6	*REEP1*	c.247delG;p.G83Afs*44	SMARD/dHMN5B	< 1		UR	UR	5.33	–	–	–	–	LP
PaC14	*MFN2*	c.334G > A;p.V112M	CMT2A2B	5	Vocal cord hoarseness	UR	1.6E−5	4.70	1.00*	0.32	− 2.76*	− 3.86*	P

1000G: 1000 Genomes Project, CMT: Charcot–Marie–Tooth disease, GERP: genomic evolutionary rate profiling score, gnomAD: Genome Aggregation Database, LP: likely pathogenic, P: pathogenic, LP: likely pathogenic, UR: unreported

^a^Reference DNA and protein sequences: *SH3TC2*: NM_024577.4 and NP_078853.2, *HK1*: NM_033498.3 and NP_277033.1, *REEP1*: NM_022912.3 and NP_075063.1, *MFN2*: NM_014874.3 and NP_055689.1

^b^Scores of PolyPhen-2 (PP2) ~ 1, MUpro (MU) < 0, PROVEAN (PRO) <  − 2.5, and Fathmm (Fath) <  − 1.5 indicate pathogenic prediction (*denotes a pathogenic prediction)

Mutations in the *SH3TC2* (MIM 608206) are implicated to the development of recessive CMT4C (MIM 601596) [18] and the relatively mild dominant mononeuropathy of the median nerve (MNMN, MIM 613353) [19]. We identified one novel and one rare homozygous variants of *SH3TC2* in two families. As the first mutation, a novel homozygous c.2599C > T mutation which results in a stop-gain mutation (p.Gln867*) was identified in a 6-year-old boy (family ID: PaC2). The *SH3TC2* mutation was heterozygous in the unaffected parents and a younger brother (Fig. 1a). This mutation has not been reported as pathogenic, nor has it been registered in the public databases of dbSNP, 1000G, or gnomAD. The p.Gln867* mutation is expected to result in the production of a truncated protein which lacks many tetratricopeptide repeat (TPR) domains. Although p.Gln867* had not been reported yet, several stop-gain mutations, such as p.Gln892*, p.Arg904*, and p.Tyr943*, have been reported as the underlying causes of CMT4C [18, 20, 21]. As the second *SH3TC2* mutation, c.3650G > A resulting in p.Gly1217Asp was identified in a 10-years-old boy (family ID: PaC3). The unaffected parents and younger sister and brother were heterozygous for the mutation (Fig. 1b). The homozygous p.Gly1217Asp mutation is still not reported as pathogenic, although the same heterozygous variant was recently registered as “uncertain significance” in the ClinVar database. It was registered in the dbSNP (rs758669363) and gnomAD with a very low allele frequency (1.6E-5). The p.Gly1217Asp mutation was located in the highly conserved TPR domain, which plays a putative role in protein–protein interactions (Fig. 2b,c), and in silico prediction analysis using PolyPhen-2, Fathmm, and PROVEAN determined it as being pathogenic.

Only a few cases with homozygous mutations in *HK1* have been reported to be associated with autosomal recessive CMT4G (MIM 605285), also called HMSN Russe type [22, 23]. An 11-year-old boy with demyelinating CMT (family ID: PaC4) harbored a stop-gain mutation. i.e., c.19C > T (p.Arg7*) in *HK1* (MIM 142600), which putatively resulted in a very short premature peptide. The affected boy was homozygous for the mutation and the unaffected father and a younger brother were heterozygous for the mutation (Fig. 1c). This *HK1* mutation was not reported as pathogenic in CMT patients. The mutation was registered in the dbSNP (rs779250530) and the gnomAD with a very low allele frequency (2.0E-5), but all reported individuals were heterozygous for the mutation.

A small number of mutations in *REEP1* (MIM 609139) have been reported to cause several neuromuscular disorders, such as the dominant dHMN5B (MIM 614751), also known as distal spinal muscular atrophy type 5B (dSMA5B) [24], and dominant spastic paraplegia-31 (SPG31, MIM 610250) [25]. A homozygous splicing mutation was also recently reported in a patient manifesting similar symptoms of spinal muscular atrophy with respiratory distress (SMARD), which has a phenotype similar to that of SMA but with an additional symptom of diaphragmatic palsy [26]. In this study, we identified a homozygous frameshift *REEP1* mutation of c.247delG in a 2.5-year-old girl with dHMN (family ID: PaC6). This deletion was expected to produce a truncated premature peptide (p.Gly83Alafs*44). The unaffected parents and an elder sister harbored a heterozygous version of this mutation (Fig. 1d). It has not been registered in any databases, nor has it been reported as a pathogenic mutation.

Most mutations in *MFN2* (MIM 608507) are associated with autosomal dominant CMT2A2A (MIM 609260) [27] and CMT6A (MIM 601152) [28], whereas, recessive *MFN2* mutations have been rarely reported with more severe and earlier onset CMT2A2B (MIM 617087) [29]. The affected 7-year-old boy in the PaC14 family harbored a homozygous c.334G > A (p.Val112Met) in *MFN2*. The unaffected parents and a younger sister harbored a heterozygous version of this mutation (Fig. 1e). The mutation was reported in the gnomAD with a very low frequency (1.6E-5), and was registered as likely pathogenic in the ClinVar database. It was predicted to be pathogenic by the in silico analysis using PolyPhen-2, Fathmm, and PROVEAN, and this mutation was found to be located in the highly conserved GTPase domain among vertebrate species (Fig. 2b,c).

Filtering WES data for the affected individuals of five families revealed several rare functionally significant variants (MAF < 0.01) in CMT-related genes, in addition to the above mentioned five pathogenic or likely pathogenic mutations (Table 3). A homozygous *DST* c.1933A > G (p.Ile645Val) variant was observed in the PaC4 patient. The *DST* mutation was cosegregated with the affected individual. However, in silico analyses using PolyPhen-2 and PROVEAN programs predicted it to be nonpathogenic. *DST* mutations have been reported to be implicated in HSAN6 (MIM 614653) [30]. Thus, we classified this homozygous variant as a ‘variant of uncertain significance (VUS)”. All other rare variants were considered as nonpathogenic because they were either nonsegregated with the affected individuals when all the family members were tested by Sanger sequencing or did not fit the inheritance modes of the corresponding genes.

**Table 3:** Rare variants observed in CMT-related genes from patients in the five Pakistani CMT families

Family	Gene	Variant		ClinVar	dbSNP151	Allele frequency		GERP	In silico analysis^a^			Note
		Nucleotide	Amino acid			1000G	gnomAD		PP2	PRO	Fath	
PaC2	*KIF1B*	c.3209C > T	p.A1070V	UR	rs768176241	UR	1.8E−05	4.82	0.02	− 0.01	− 0.77	Nonsegregation, LB
	*DST*	[c.7252G > A + c.7765A > G]	[p.V2418I + p.I2589V]	UR	rs62621210	0.0400	0.0360	4.80	0.00	0.76	1.25	*Cis*, nonsegregation, LB
				B,LB	rs150191284	0.0102	0.0249	4.45	0.02	0.07	− 0.74	
	*MYH14*	c.3748G > T	p.V1250L	LB	rs202242879	0.0006	0.0007	3.78	0.11	− 0.39	− 1.17	Nonsegregation, LB
	*SCN11A*	c.1732 T > A	p.F578I	UR	rs772393665	UR	7.1E−05	5.58	0.98*	− 4.61*	− 4.30*	Nonsegregation, LB
PaC3	*KIF1B*	[c.2107 T > C] + [c.2455A > C]	[p.W703R] + [p.S819R]	B,LB	rs551543997	0.0054	0.0026	5.32	0.99*	− 10.0*	− 0.93	*Trans*, nonsegregation, LB
				LB	rs140015591	0.0002	0.0003	− 2.96	0.02	− 2.19	− 1.03	
	*NTRK1*	[c.2339G > A + c.2360C > T]	[p.R780Q + p.A787V]	B,LB	rs35669708	0.0038	0.0046	4.10	0.87*	− 1.25	− 1.60*	*Cis*, nonsegregation, LB
				UR	rs761207548	UR	5.0E−05	4.07	0.36	− 3.00*	− 1.13	
	*NAGLU*	c.2209C > A	p.R737S	B	rs86312	0.0116	0.0183	4.01	0.47*	− 0.19	− 5.34*	Nonsegregation, LB
	*SCN10A*	c.3887G > T	p.S1296I	LB	rs779527264	UR	0.0002	4.88	1.00*	− 5.79*	− 4.55*	Nonsegregation, LB
PaC4	*DST*	[c.1933A > G] + [c.1933A > G]	[p.I645V] + [p.I645V]	UR	rs754692637	UR	8.0E−06	2.51	0.00	− 0.20	− 0.81	Homozygous, cosegregation, VUS
	*ARHGEF10*	c.2566G > A	p.V856I	UR	rs773521162	UR	0.0003	4.25	0.87*	− 0.62	0.15	Nonsegregation, LB
PaC6	*TFG*	c.175A > G	p.K59E	UR	rs1232918261	UR	UR	5.90	1.00*	− 3.02*	− 3.02*	Nonsegregation, LB
PaC14	*SETX*	c.2385_2387delTTT	p.I795_K796delinsM	UR	rs755971927	UR	5.0E−05	–	–	–	–	Nonsegregation, LB

1000G: 1000 Genomes Project, B: benign, gnomAD: Genome Aggregation Database, LB: likely benign, VUS: variant of uncertain significance, *trans*: *trans* arrangement of variants in homologous chromosomes (bi-alleles), *cis*: *cis* arrangement of variants in a chromosome, UR: unreported

aScores of PolyPhen-2 (PP2) ~ 1, PROVEAN (PRO) < − 2.5, and Fathmm (Fath) < − 1.5 indicate pathogenic prediction (* denotes a pathogenic prediction)

### Homozygosity mapping

**Fig. 3:**
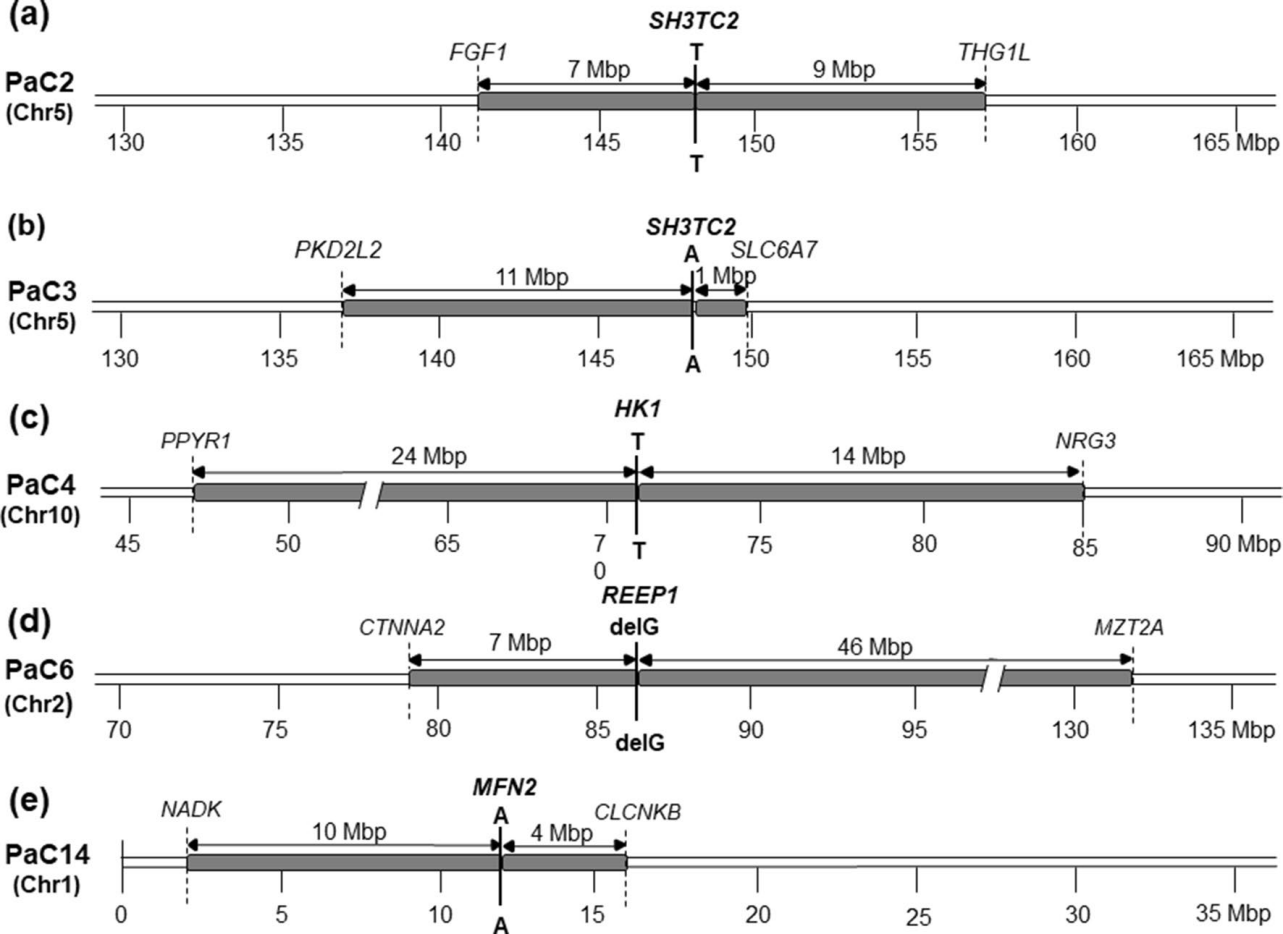
Homozygosity mapping of the chromosomal regions around the pathogenic mutations for the affected individuals from the consanguineous Pakistani CMT families. Genes and SNP numbers located at the ends of the homozygous blocks (HBs) and their approximate chromosomal positions (indicating by Mbp) are shown at the top and bottom of the maps, respectively. **a** 16 Mbp HB from *FGF1* to *THG1L* in the PaC2 family with *SH3TC2* mutation. **b** 12 Mbp HB from *PKD2L2* to *SLC6A7* in the PaC3 family with *SH3TC2* mutation. **c** 38 Mbp HB from *PPYR1* to *NRG3* in the PaC4 family with *HK1* mutation. **d** 53 Mbp HB from *CTNNA2* to *MZT2A* in the PaC6 family with *REEP1* mutation. **e** 14 Mbp HB from *NADK* to *CLCNKB* in the PaC14 family with *MFN2* mutation

We observed homozygous blocks (HBs) at the chromosomal regions including pathogenic or likely pathogenic mutations in all the five affected individuals by the SNP haplotype analysis using WES data (Fig. 3). The lengths of the HBs were approximately 16 Mbp from *FGF1* (rs34003) to *THG1L* (rs2270812) in the PaC2 family with *SH3TC2* mutation, 12 Mbp from *PKD2L2* (rs700605) to *SLC6A7* (rs12653451) in the PaC3 family with *SH3TC2* mutation, 38 Mbp from *PPYR1* (rs1936339) to *NRG3* (rs478010) in the PaC4 family with *HK1* mutation, 53 Mbp from *CTNNA2* (rs2228460) to *MZT2A* (rs10182785) in the PaC6 family with *REEP1* mutation, and 14 Mbp from *NADK* (rs7407) to *CLCNKB* (rs7517792) in the PaC14 family with *MFN2* mutation. Considering the consanguinity in the families, these HBs suggest that both homozygous alleles in each family originated from a single ancestor.

## Discussion

In this study, we performed genetic screening in consanguineous Pakistani CMT families by WES and identified five homozygous mutations in four genes, *SH3TC2, HK1, REEP1*, and *MFN2*, as the underlying cause of recessive CMT. The mutations identified in this study have not been reported in CMT patient, thus far.

*SH3TC2*, which encodes an SH3 domain and tetratricopeptide repeats-containing protein 2, is expressed in Schwann cells of peripheral nerves, suggesting a possible role in myelination [31]. Mutations in *SH3TC2* cause recessive CMT4C usually concurrent with scoliosis, with the onset ranging from infancy to early teens [18]; however, cases with late onset (≤ 30 years) were also reported [32].

Mutations in *HK1* cause recessive CMT4G (HMSNR), mostly found in the Spanish Gypsy patients [22]. Hexokinase 1 encoded by *HK1* catalyzes the phosphorylation of glucose. HK1 localizes at the outer membrane of mitochondria (OMM) through a porin-binding domain, and it was suggested that non-OMM-binding HK1 proteins involve in the pathogenesis of CMT4G pathogenesis [22]. Several *HK1* mutations are also associated with autosomal dominant retinitis pigmentosa-79 (RP79, MIM 617460), which exhibits variable phenotype with ages of onset ranging from childhood to 70 years [33]. The affected 11-year-old boy with the *HK1* mutation did not manifest symptoms of retinitis pigmentosa until his age of examination.

*REEP1* encodes a receptor accessory protein 1, suggesting its role in facilitating endoplasmic reticulum (ER)-mitochondrial interactions [34]. Reep1 null mice displayed alterations in ER morphogenesis and lipid abnormalities [35]. It is known that the *REEP1* mutations exhibited considerable phenotypic heterogeneity [36]. Dominant *REEP1* mutations have been reported to cause dHMN5B (DSMA5B) and SPG21 with the onset ages falling in either the first or second decades [24, 25]. Recently, a recessive *REEP1* mutation (p.Phe62Lysfs*23) was reported in a 5-year-old Lebanese boy manifesting a similar SMARD phenotype [26]. The affected boy presented foot deformity and contractures of the distal phalanges at the time of birth. This was similar to PaC6 family who harbored a p.Gly83Alafs*44 mutation resulting in a premature termination and exhibited a similar onset age, although some clinical features were different. In the nerve conduction studies, none of the motor nerves were evoked, but all sensory nerves showed normal SNAPs and SNCVs. From the clinical and NCV findings, this patient's symptoms are apparently similar to those of SMARD.

*MFN2* encodes mitofusin 2 which plays an important role in maintaining equilibrium between mitochondrial fusion and fission [37]. Most *MFN2* mutations have been reported to cause dominant CMT2A2A [27]. However, some homozygous or compound heterozygous mutations cause recessive CMT2A2B (MIM 617087) that is associated with more severe and earlier onset phenotypes. Nicholson et al. suggested that CMT2A2B may be semidominant, and carriers harboring a single mutant allele may manifest a weak phenotype with incomplete penetrance [29]. Our patient harboring homozygous *MFN2* mutation manifested relatively early onset (5 years old) and severe phenotypes, corresponding to CMT2A2B phenotypes. Additionally, vocal cord paralysis seen in the affected boy has been occasionally reported in CMT2A patients harboring *MFN2* mutations [38, 39]. The patient showed no symptom of optic atrophy which is a characteristic of CMT6A caused by *MFN2* mutations until the time of the first examination (7 years old). His parents harboring the heterozygous *MFN2* mutation were apparently unaffected; however, clinical and electrophysiological tests were not performed. Therefore, it is likely that they might be mildly affected because of the semidominant nature of the mutation.

Next generation sequencing data can be used for homozygosity mapping of recessive traits in consanguineous pedigrees [40]. When we performed homozygosity mapping around the pathogenic mutations using SNPs from the WES data of the affected members, all the examined families showed same haplotype blocks between two homologous chromosomes. This suggests that both alleles of the homozygous mutations identified in each family originated from a single ancestor. Although a few CMT cases were investigated in this study, the frequency of recessive patients with homozygous mutations was certainly higher than that in other countries, such as United Kingdom, United States, Italy, and Japan [5, 41, 42]. Mutations in *MFN2* are well recognized as the cause of dominant CMT2; however, recessive homozygous mutations have been rarely reported.

## Conclusions

In conclusion, we identified five pathogenic or likely pathogenic mutations in consanguineous Pakistani families with early onset CMT. All the mutations were novel, and the genotype–phenotype correlations were confirmed. We believe that our findings will expand our understanding of the genetic basis of peripheral neuropathy and improve molecular diagnostics and treatment options.

## Data Availability

The datasets generated and analyzed during the current study are available in the NCBI Sequence Read Archive Database (Accession No.: PRJNA735898; https://www.ncbi.nlm.nih.gov/Traces/study/?acc=PRJNA735898). The web links of the relevant datasets were as follows: hg19 (http://genome.ucsc.edu/), 1000 Genomes project (http://www.1000genomes.org/), dbSNP (http://www.ncbi.nlm.nih.gov/snp), gnomAD (https://gnomad.broadinstitute.org/about), ClinVar (https://www.ncbi.nlm.nih.gov/clinvar/), and OMIM (http://omim.org).

## Abbreviations

ACMG: American College of Medical Genetics and Genomics
CMAP: Compound muscle action potential
CMT: Charcot–Marie–Tooth disease
dHMN: Distal hereditary motor neuropathy
dSMA5B: Distal spinal muscular atrophy type 5B
ER: Endoplasmic reticulum
FDS: Functional disability scale
GERP: Genomic evolutionary rate profiling score
HB: Homozygous block
HMSN: Hereditary motor and sensory neuropathy
HSAN: Hereditary sensory and autonomic neuropathy
MAF: Minor allele frequency
MNCV: Motor nerve conduction velocity
NCV: Nerve conduction velocity
OMM: Outer membrane of mitochondria
SMARD: Spinal muscular atrophy with respiratory distress
SNAP: Sensory nerve action potential
SNCV: Sensory nerve conduction velocity
TPR: Tetratricopeptide repeat
WES: Whole exome sequencing
